# Hydraulic strategy defines contrasting responses to an abrupt precipitation during a successive lethal drought

**DOI:** 10.1186/s12870-024-05859-y

**Published:** 2024-11-29

**Authors:** Xiaoying Lin, Caixiao Wu, Kaikai Zhang, Haoran Dong, Ling Xiao, Fan Li, Yao Huang, Qiang Li

**Affiliations:** 1 NHC Key Laboratory of Tropical Disease Control, School of Tropical Medicine, Hainan Medical University, Haikou, 571199 China; 2 Ministry of Education Key Laboratory for Genetics and Germplasm Innovation of Tropical Special Forest Trees and Ornamental Plants, Hainan University, Haikou, 570228 China

**Keywords:** Iso/anisohydric, Mortality, Non-structural carbohydrates, Recovery

## Abstract

**Background:**

As precipitation patterns are predicted to become more erratic, it’s vital to understand how abrupt climate events will affect woody seedlings that develop different hydraulic strategies. We cultivated anisohydric *Robinia pseudoacacia* L. and isohydric *Quercus acutissima* Carr. in a greenhouse, and subjected an abrupt precipitation event during a successive drought. Patterns of leaf and root gas exchange, leaf and stem hydraulics, seedlings growth, and non-structural carbohydrate (NSC) patterns were determined.

**Results:**

We found that as an anisohydric species, *R*. *pseudoacacia* seedlings adopted a strategy of sacrificing leaves in response to stress, resulting in the lowered photosynthesis and ultimately leading to a decrease in NSC accumulation. In contrast, isohydric *Q*. *acutissima* maintained the integrity of leaves by reducing respiratory consumption in response to drought stress, thereby ensured the stability of NSC pool.

**Conclusion:**

*R*. *pseudoacacia* exhibited an extravagant strategy with efficient water transport, photosynthetic assimilation, and growth capabilities, but its resistance to embolism was relatively weak, while *Q*. *acutissima* adopted a resource-saving strategy with higher hydraulic safety. We also found that *Q*. *acutissima* seedlings were prone to allocate carbohydrates to maintain growth, while *R*. *pseudoacacia* preferred to sacrifice growth and aboveground NSC limitation only happened when precipitation was subjected after total stomatal closure. We thus believe that hydraulic strategy could define seedlings responses to drought and recovery, and further may adversely affect their re-sprouting capacity after drought stress relief.

## Introduction

Global climate change in the 21st century is already resulting in extreme events, and it is predicted that abrupt climate fluctuations will become more unstable in the future [27]. The abrupt change trend will further aggravate the occurrence of precipitation extremes, posing a serious threat to the stability and sustainable development of the forest ecosystem (Ahmed et al., 2017, [6]). These fluctuating events can significantly affect the microclimate or hydrological conditions in managed forests, and trigger potential effects on the structure and function of forests [2]. To evaluate how future plant communities may be affected by climatic fluctuations, we must have a comprehensive understanding of plant functional strategies in response to water stress [12]. Temperate forests are also susceptible to these extreme events considering the history of a spring drought, followed by abundant and rapid precipitations in summer, resulting in a significant fluctuation in soil moisture [13]. There is still a knowledge gap on how the woody plants in warm temperate forests adapt and respond to the abrupt precipitation fluctuations. Especially, the seedlings are more sensitive to various water stress, calling for our attention to plant hydraulic strategies (Michael et al., 2023).

Plant traits in response to drought may contribute to improving their adaptability to climate change [25]. Plants can adopt different degree of stomatal sensitivity in response to water condition changes, forming a continuous spectrum of isohydric-anisohydric. Isohydric plants show conservation in water use, maintaining the water potential relatively stable, while anisohydric plants adopt a strategy of "water consumer", experiencing water potential fluctuations [16]. The regulation of continuous water status is regarded as a key factor in the regulation of plant survival under drought conditions. Isohydric species would reduce stomatal conductance and CO_2_ uptake during drought, and thus are more likely to suffer from carbon starvation [5, 30]. However, when more severe embolism occurs in the xylem of anisohydric species, the crown of plants may dieoff due to hydraulic failure, eventually leading to whole-plant death [23, 25].

Plant functional strategies in response to water fluctuations involves the integration of multiple complex processes that interact with biological and abiotic factors, so it is difficult to attribute them to a single attribute. Studies carried out under controlled experimental conditions could help us to explore plant responses to stressed conditions and analyze the general effects of abrupt climate events more precisely. Based on the species distribution patterns as well as previous relevant findings, this study selected an anisohydric species *Robinia pseudoacacia* L. and an isohydric species *Quercus acutissima* Carr., which are the predominant and widely distributed species in warm temperate regions [7]. *R*. *pseudoacacia*, an alien tree species of the legume family, introduced into China about 200 years ago, is now widely mixed with *Q*. *acutissima*, the original tree species of the Fagaceae family [19, 20]. Relevant investigation results show that about 15 provinces and cities in China have begun to plant and cultivate *R*. *pseudoacacia*. Due to the resistance to dry and cool climate, *R*. *pseudoacacia* is suitable for drought and semi-arid areas, and is widely planted in northern China and loess areas in northwest China, with remarkable ecological benefits [9]. *Q*. *acutissima* usually grows in coniferous and hardwood forests in temperate mountainous and hilly areas, and is widely distributed in northern China [18]. Our previous study showed that the anisohydric *R*. *pseudoacacia* seedlings are prone to die of hydraulic failure, and isohydric *Q*. *acutissima* seedlings are more carbon limited under water-limited soil conditions [17].

Here, we selected *R*. *pseudoacacia* and *Q*. *acutissima* that were defined contrasting in hydraulic strategies. We detected their responses to a manipulated abrupt precipitation, and their carbon allocation patterns after seedlings mortality during a successive drought. We hypothesized that (i) according to their hydraulic strategies, *R*. *pseudoacacia* seedlings could recover faster than *Q*. *acutissima* after an abrupt precipitation; (ii) as a fast-growing species, *R*. *pseudoacacia* seedlings were prone to allocate more carbon to maintain growth, while *Q*. *acutissima* were more likely to constraint growth to save carbon.

## Materials and methods

### Plant material and experimental design

This greenhouse experiment was conducted during summer at Fanggan Research Station in Shandong Province, China (36.43° N, 117.45° E). In the previous autumn, seeds of *Q*. *acutissima* were collected near the station and seeds of *R*. *pseudoacacia* were purchased from Qiluyuanyi Seed Company (Linyi, China). In the spring, the seeds were germinated in deionized water and were then transferred to a plastic pot (32 cm in depth and 29 cm in diameter) with an 8 kg growth substrate. The plant growth matrix was composed of air-dried sandy loam soil and humus soil at a ratio of 2:3 in volume, and was fertilized biweekly with slow-release fertilizer. Air temperature, relative humidity, and photosynthetic active radiation photo flux density (PPFD) were measured every 10 min using the HOBO data recorder (U12-012, Onset, Bourne, MA, USA). During the experiment, the average greenhouse environmental conditions were as follows: the daytime temperature was 32℃, the night temperature was 21℃, the daytime relative humidity was 60%, and the night relative humidity was 95%. Maximum PPFD was above 1600 μmol m ^−2^ s ^−1^.

**Fig. 1:**
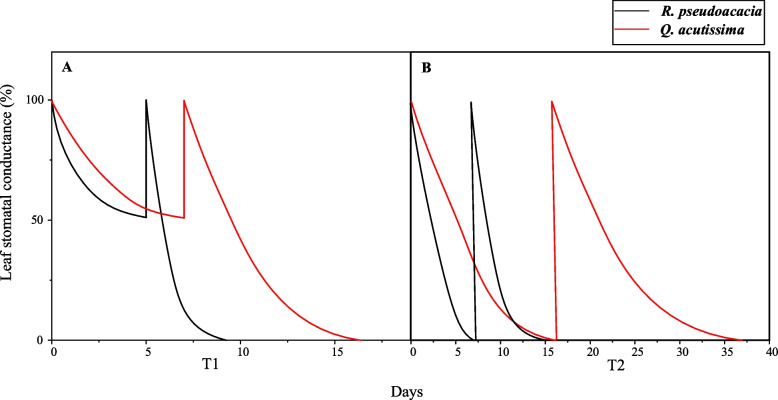
Scheme of abrupt precipitation manipulations

A complete block design was applied, where 40 seedlings for both species were randomly assigned (*n* = 10) and randomly distributed in the greenhouse. The control group was well-hydrated daily. Two treatments were applied to manipulate abrupt precipitations during a successive drought process, according to the daily measured leaf stomatal conductance (*g*_s_) using a portable steady-state leaf porometer (SC-1, Decagon devices Inc., Pullman, WA, USA). Specifically, the first treatment (T1) of abrupt precipitation manipulation was applied when the average value of the *g*_s_ declined to 50% of the well-watered control, and the second abrupt precipitation treatment (T2) was manipulated when the average g_s_ value no longer declined for 3 days (near total stomatal closure). Twenty-four hours after manipulated precipitations, we randomly selected 5 seedlings from each group for recovery detection, and the remaining 5 individuals were kept droughted on until seedlings mortality (Fig. 1). Seedlings were considered dead when the foliar turned dry or become detached from petioles and the stems appeared brown. We detected the seedlings recovery by quantifying leaf damage, and measured leaf gas exchanges, leaf and stem water potentials, stem hydraulic conductivity, and root respiration. After mortality, seedlings were harvested for carbohydrate analyses.

### Leaf shedding and growth determination

To assess the degree of leaf discoloration and shedding, we used biomass estimation methods. Specifically, we collected the littered and discolored leaves 24 h after the manipulated precipitations applied, and then oven-dried them for dry weight determination. After seedlings harvest and the above-ground biomass determined, the degree of leaf discoloration and shedding was calculated. Before biomass measurement, root respiration rate was measured.

We measured the plant heights and basal diameters at both the start and end of the treatments. The growth data were presented as the differences between the end and start.

### Gas exchange

Leaf gas exchange measurements were performed on mature and healthy leaves. An infrared gas analysis system (Li-6800, Li-Cor, Lincoln, NE, USA) was used to accurately measure gas exchange and fine root respiration. The measurements were carried out from 9:00 to 11:00 in the morning with a CO_2_ concentration of 400 μmol mol^−1^, temperature of 28℃ and humidity of 50%. When measuring net photosynthetic CO_2_ uptake (*A*_sat_) and respiratory CO_2_ emissions (*R*_root_), humidity was kept stable.

The root sample was collected and treated as follows: First, the root growing soil substrate was gently cleaned with running water and then dried with paper towels. Roots with light color, succulent and unsuberized rootlets were selected and respiration measurements were performed within 5–10 min to minimize the effect of cutting on respiratory activity [17]. During the experiment, root samples were placed in a measurement chamber to monitor the root respiration rate until the amount of carbon dioxide released remained stable. Within 2 min, a stable reading for gas exchange measurement could be obtained in a measuring chamber (6 cm^−2^), and *A*_sat_ and *R*_root_ were expressed per area.

### Water potential

The leaf water potential (Ψ_leaf_) was measured at midday (12:00–13:00 h). Meanwhile, we covered the leaf with aluminum foil for 1 h to get the balanced midday stem water potential (Ψ_stem_). all the water potential measurements were conducted with a pressure chamber (1505D-EXP, PMS Instrument, Albany, OR, USA).

### Stem hydraulics

Before the hydraulic conductivity measurements, the maximum vessel length of the five main stems for both species was estimated according to the research results of Zimmermann and Jeje (1981). The stem segments used for conductivity measurements were twice longer than the maximum vessel length. The segments were cut near the base of the stem and then recut underwater. During the experiment, the cut stem fragments were connected to a self-made hydraulic apparatus using a degassing filtered 0.5 mmol^−1^ potassium chloride solution [10]. A 60 cm hydraulic head was used to generate hydrostatic pressure to drove water through the pipe. A graduated pipette was connected to the downstream end of the stem segment for volumetric measurement of flow.

The hydraulic conductivity (*K*_s_, kg m h ^−1^ MPa ^−1^) was calculated by:

$$Kh = Jv/\left( {\Delta P/\Delta L} \right)$$

where Jv represents the flow rate in the stem fragment (kg h^−1^) and ΔP/ΔL represents the pressure gradient through the stem fragment (MPa m^−1^) [10].

### Carbohydrate analysis

After each harvest, leaf, stem, and root materials were oven-dried at 65 ℃ for 48 h for a constant weight. Then the dried sample was ground into a fine powder, which was sealed and stored in a dark environment to ensure sample stability until NSCs content was determined. For the determination of NSCs content, we referred to Hansen and Møller's [11] method. The sample was first mixed with a solution of 80% (v:v) aqueous methanol and incubated with anthrone reagent to extracted the soluble sugar (SS). Subsequently, absorbance at a wavelength of 625 nm was measured in a spectrophotometer (UV-9000 S, Metash, Shanghai, China) to assess soluble sugar content. And then the starch (St) content was determined by conversing starch to soluble substance using perchloric acid. And then the absorbance at 625 nm was measured within 30 min. To calculate the content of NSCs for each organ, SS and St was summed up.

### Statistical analysis

The means of data were compared between control and T1/T2 group with *t*-tests, before which the normality and homogeneity were assessed. The *t*-test analyses were conducted using the SPSS 23.0 software package (SPSS Inc., Illinois, USA), and the critical *ɑ*-value was set at 0.05.

## Results

### Changes in leaf surface

**Fig. 2:**
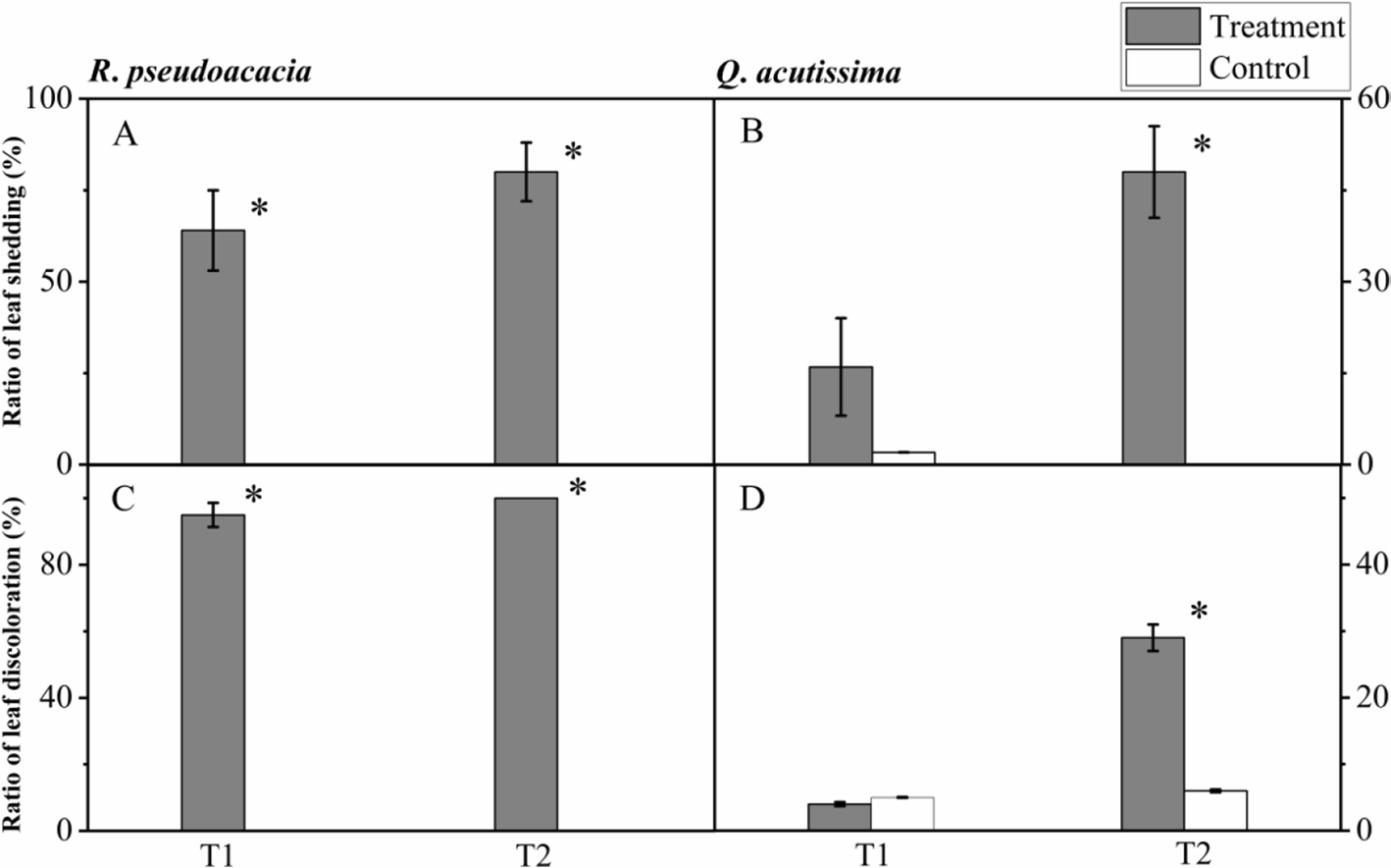
Leaf shedding and discoloration of *R. pseudoacacia* (**A**, **C**) and *Q. acutissima* (**B**, **D**) after 24 h of irrigation (T1, abrupt precipitation manipulation was applied when the average value of the *g*_s_ declined to 50% of the well-watered control; T2, abrupt precipitation treatment was applied near total stomatal closure). Data are mean ± SE; *n* = 5. Asterisks indicate significant differences between the treatments and controls (*P* < 0.05)

For *R. pseudoacacia* seedlings, the mean ratio of leaf shedding in T1 and T2 treatment was 64% ± 11% and 80% ± 8.0% respectively, and significantly different with control, as there was no leaf exfoliated in the control group (Fig. 2A). Besides, the mean ratio of leaf discoloration in T1 treatment was 95% ± 3.6%, and all the leaves in T2 treatment were discolored, and both significantly different with control, as there was no leaf discoloration in the control group (Fig. 2C).

For *Q. acutissima* seedlings, neither leaf discoloration nor leaf shedding was significantly different between T1 and C1 (Fig. 2B), but both of them were significantly different between T2 and C2 (Fig. 2D). Specifically, the mean ratio of leaf shedding in T2 treatment was 48% ± 7.5%, but there was no leaf exfoliated in the control group (Fig. 2B). And the mean ratio of leaf discoloration in T2 treatment and control was 29% ± 2.0% and 4.0% ± 0.3% respectively (Fig. 2D).

### Gas exchange

**Fig. 3:**
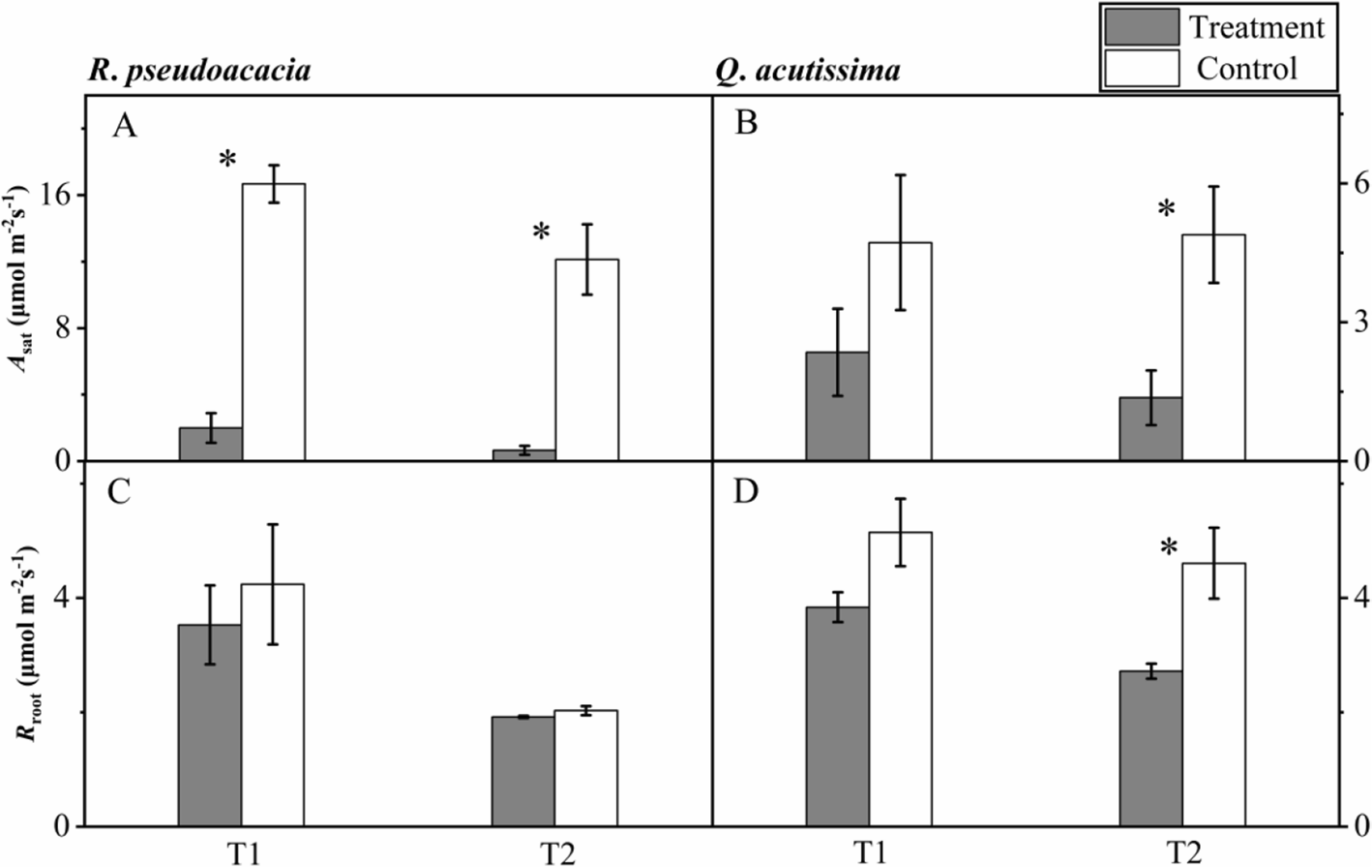
Leaf net photosynthesis (*A*_sat_) and root respiration (*R*_root_) of *R. pseudoacacia* (**A**, **C**) and *Q. acutissima* (**B**, **D**) after 24 h of irrigation (T1, abrupt precipitation manipulation was applied when the average value of the *g*_s_ declined to 50% of the well-watered control; T2, abrupt precipitation treatment was applied near total stomatal closure). Data are mean ± SE; *n* = 5. Asterisks indicate significant differences between the treatments and controls (*P* < 0.05)

For *R. pseudoacacia* seedlings, leaf net photosynthesis differed significantly both between T1 and C1, and between T2 and C2. Specifically, the mean *A*_sat_ in T1 treatment and control was 1.99 ± 0.89 and 16.67 ± 1.12 µmol m ^−2^ s ^−1^ respectively, and that in T2 treatment and control was 0.65 ± 0.26 and 12.13 ± 2.12 µmol m ^−2^ s ^−1^ respectively (Fig. 3A). But the root respiration was not significantly different between T1 and C1, either between T2 and C2 (Fig. 3C).

For *Q. acutissima s*eedlings, there was no significant difference between T1 and C1 either in leaf net photosynthesis or in root respiration (Fig. 3B), but there was significant difference between T2 and C2 both in leaf net photosynthesis and in root respiration (Fig. 3D). Specifically, the mean *A*_sat_ in T2 treatment and control was 1.37 ± 0.59 and 4.89 ± 1.04 µmol m ^−2^ s ^−1^ respectively, and the mean *R*_root_ in T2 treatment and control was 2.72 ± 0.13 and 4.61 ± 0.62 µmol m ^−2^ s ^−1^ respectively.

### Plant water status

**Fig. 4:**
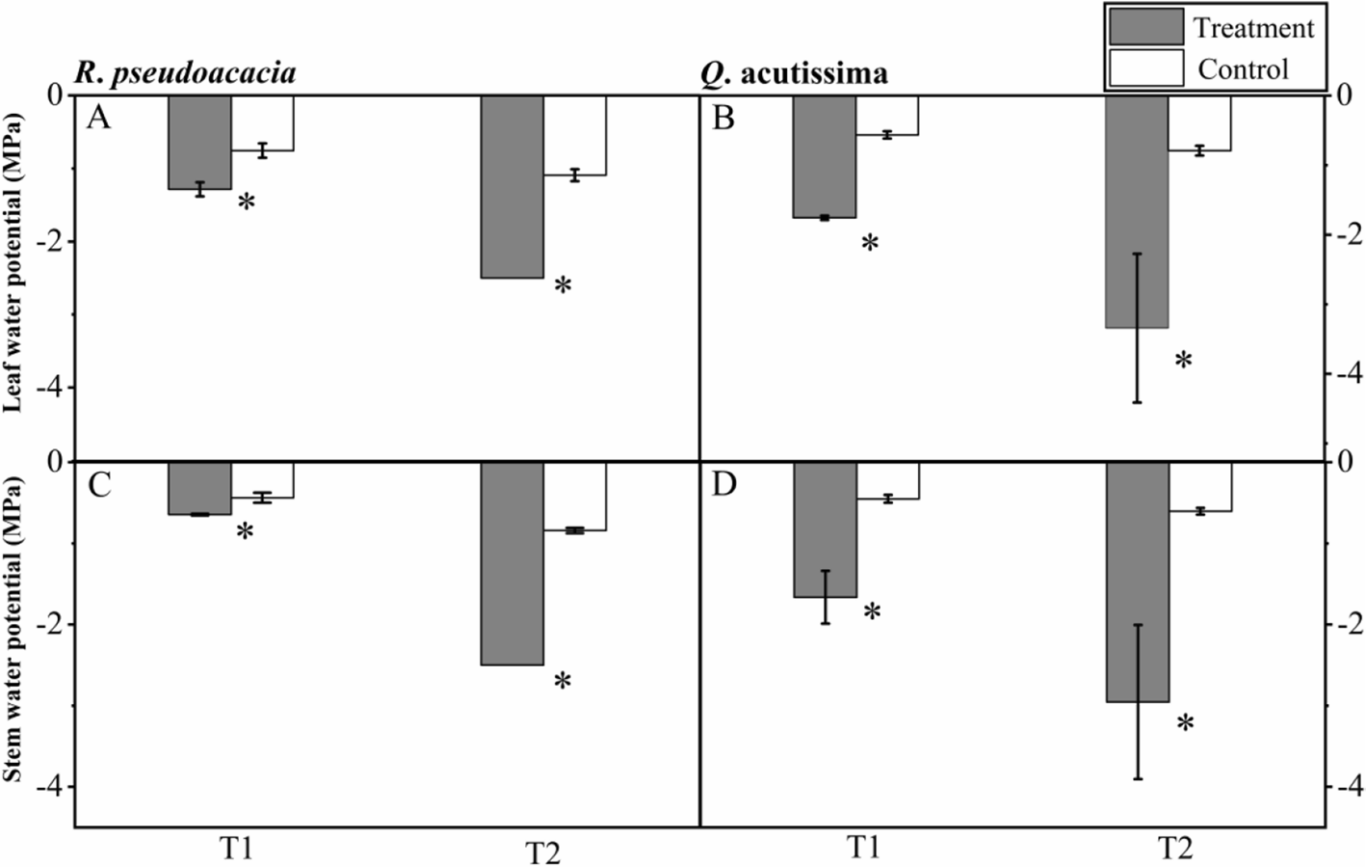
Leaf and stem water potentials of *R. pseudoacacia* (**A**, **C**) and *Q. acutissima* (**B**, **D**) after 24 h of irrigation (T1, abrupt precipitation manipulation was applied when the average value of the *g*_s_ declined to 50% of the well-watered control; T2, abrupt precipitation treatment was applied near total stomatal closure). Data are mean ± SE; *n* = 5. Asterisks indicate significant differences between the treatments and controls (*P* < 0.05)

For *R. pseudoacacia* seedlings, both leaf and stem water potentials were significantly different between T1 and C1, as well as T2 and C2. Specifically, the mean leaf water potential in T1 treatment and control was −1.28 ± 0.10 and −0.75 ± 0.1 MPa respectively, and that in T2 treatment and control was −2.50 ± 0 and −1.09 ± 0.08 MPa respectively (Fig. 4A). The mean stem water potential in T1 treatment and control was −0.65 ± 0.01 and −0.44 ± 0.06 MPa respectively, and that in T2 treatment and control was −2.50 ± 0 and −0.84 ± 0.03 MPa respectively (Fig. 4C).

For *Q. acutissima* seedlings, both leaf and stem water potential patterns were same as *R. pseudoacacia* seedlings. Specifically, the mean leaf water potential in T1 treatment and control was −1.75 ± 0.03 and −0.56 ± 0.05 MPa respectively, and that in T2 treatment and control was −3.34 ± 1.07 and −0.79 ± 0.07 MPa respectively (Fig. 4B). The mean stem water potential in T1 treatment and control was −1.66 ± 0.33 and −0.45 ± 0.05 MPa respectively, and that in T2 treatment and control was −2.95 ± 0.95 and −0.60 ± 0.04 MPa respectively (Fig. 4D).

**Fig. 5:**
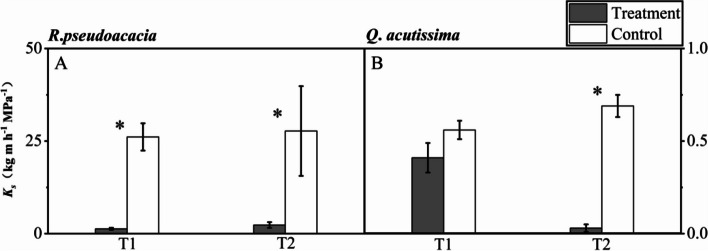
Specific stem hydraulic conductivity (*K*_*s*_) of *R. pseudoacacia* (**A**, **C**) and *Q. acutissima* (**B**, **D**) after 24 h of irrigation (T1, abrupt precipitation manipulation was applied when the average value of the *g*_s_ declined to 50% of the well-watered control; T2, abrupt precipitation treatment was applied near total stomatal closure). Data are mean ± SE; *n* = 5. Asterisks indicate significant differences between the treatments and controls (*P* < 0.05)

For *R. pseudoacacia* seedlings, there were significant difference both between T1 and C1, and T2 and C2 in *K*_*s*_. Specifically, the mean *K*_*s*_ in T1 treatment and control was 1.31 ± 0.29 and 26.13 ± 3.69 kg m h ^−1^ MPa ^−1^ respectively, and that in T2 treatment and control was 2.34 ± 0.77 and 27.72 ± 6.11 kg m h ^−1^ MPa ^−1^ respectively (Fig. 5A).

For *Q. acutissima* seedlings, there was no significant difference between T1 and C1 in *K*_*s*_, but not that was significantly different between T2 and C2, where the mean *K*_*s*_ in T2 treatment and control was 0.03 ± 0.02 and 0.69 ± 0.06 kg m h ^−1^ MPa ^−1^ respectively (Fig. 5B).

### Contents of non-structural carbohydrates after seedlings mortality

**Fig. 6:**
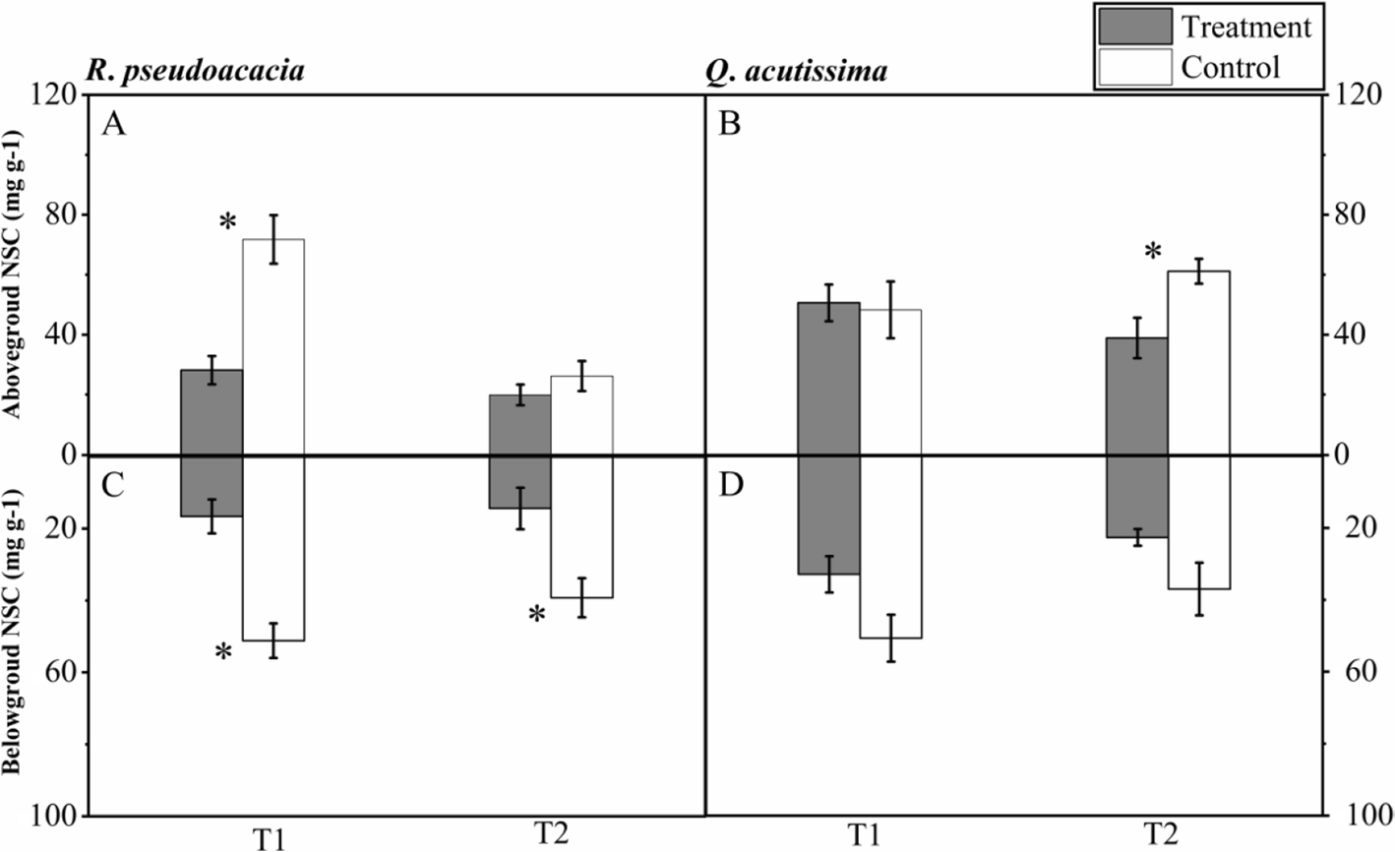
Aboveground and belowground non-structural carbohydrates of *R. pseudoacacia* (**A**, **C**) and *Q. acutissima* (**B**, **D**) after seedlings mortality (T1, abrupt precipitation manipulation was applied when the average value of the *g*_s_ declined to 50% of the well-watered control; T2, abrupt precipitation treatment was applied near total stomatal closure). Data are mean ± SE; *n* = 5. Asterisks indicate significant differences between the treatments and controls (*P* < 0.05)

For *R. pseudoacacia* seedlings, there were significant differences between T1 and C1 both in aboveground and belowground NSC content. Specifically, the mean aboveground NSC content in T1 treatment and control was 28.17 ± 4.74 and 71.78 ± 8.10 mg g ^−1^ respectively, and belowground NSC content was 16.41 ± 4.72 and 51.18 ± 4.83 mg g ^−1^ respectively (Fig. 6A, C). Besides, only belowground NSC content differed significantly between T2 and C2, 14.40 ± 5.78 and 39.23 ± 5.42 mg g ^−1^ respectively (Fig. 6C).

For *Q. acutissima* seedlings, there was no significant differences either in aboveground or belowground NSC content between T1 and C1. And there was only significant difference in aboveground NSC content between T2 and C2, where the mean aboveground NSC content in T2 treatment and control was 38.89 ± 6.76 and 61.16 ± 4.10 mg g ^−1^ respectively (Fig. 6B).

### Growth indicators

**Fig. 7:**
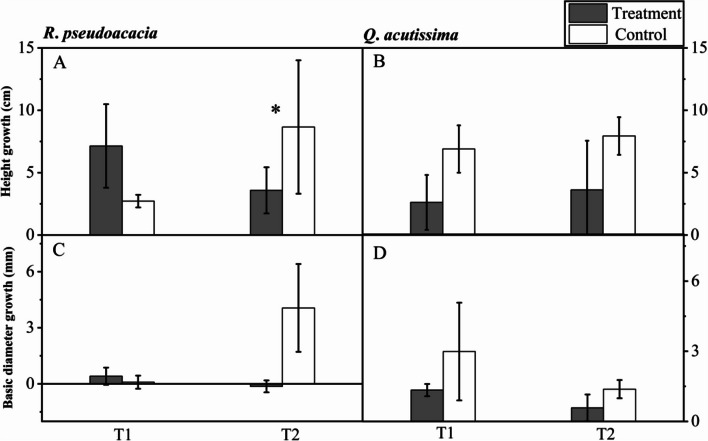
Height and basal diameter growth of *R. pseudoacacia* (**A**, **C**) and *Q. acutissima* (**B**, **D**) after seedlings mortality (T1, abrupt precipitation manipulation was applied when the average value of the *g*_s_ declined to 50% of the well-watered control; T2, abrupt precipitation treatment was applied near total stomatal closure). Data are mean ± SE; *n* = 5. Asterisks indicate significant differences between the treatments and controls (*P* < 0.05)

For *R. pseudoacacia* seedlings, there was only significant difference in height growth between T2 and C2, 3.58 ± 0.85 and 8.66 ± 2.35 cm respectively (Fig. 7A). For *Q. acutissima* seedlings, there was no significant difference in growth (Fig. 7B, D).

## Discussion

Our study showed that seedlings of isohydric *Q. acutissima* and anisohydric *R. pseudoacacia* responded differently to the abrupt precipitation we manipulated, and the hydraulic strategy could further define their carbohydrate allocation patterns when a successive drought leading to seedlings death.

### Short-term recovery in response to a manipulated precipitation

After rehydration, the seedlings of *R. pseudoacacia* sacrificed the leaves (either via shedding or discoloration), while *Q. acutissima* recovered without obvious leaf loss or color changes. From the perspective of construction cost, cheap leaves are often preferentially sacrificed under stress to protect stems and roots, the tissues that are more carbon-cost. Leaf wilting and shedding is actually an important adaptive mechanism for plants in face to abiotic stress. This mechanism plays a crucial role in inhibiting transpiration, effectively reducing water loss or improving water use efficiency, and delaying the occurrence of lethal stress [4, 36]. Meanwhile, leaf wilting and shedding could help osmoregulation and adjust cell wall elasticity, thus reaching the turgor loss point at low water potential and relative water content to undergo drought stress [1]. The natural shedding process of leaves provides the remaining active leaves to use the limited water and mineral resources, and also helps to avoid the decline of water conductivity caused by cavitation, thus ensuring the overall growth and physiological stability [8]. In view of this strategy, hydraulic failure is effectively controlled in the distal region (leaf), thus ensuring that the stem maintains the hydraulic conductivity during the early stage of drought [15]. Besides, the carbohydrates stored in the leaves cannot meet the need to maintain their water state above the critical water potential threshold. Therefore, the older leaves are gradually dehydrated due to hydraulic failure, which leads to the leaf drying and death eventually [26]. Conversely, *Q. acutissima* seedlings adopted a conservative strategy to maintain leaf integrity during drought stress, which might be associated with their higher leaf construction cost (indicated by higher specific leaf area) [31]. Moreover, the isohydric species (that is, *Q. acutissima*) are more tolerant of drought stress, profited by timely stomatal closure [17], osmotic adjustment (Meinzer et al., 2016; [21]), and rapidly-recovered conduits (Fig. 5B).

Besides, the seedlings of *Q. acutissima* inhibited the root respiration in response to rehydration. In the face of drought stress, roots usually generate chemical signals and transmit these signals upward to trigger a timely stomatal closure [24]. At the same time, plant roots will also adjust to cope with the changes of surrounding soil water, such as architecture changes, inhibited root respiration (Fig. 3D) to weaken the metabolic activities relating to plant growth, especially when the carbon assimilation was restrained (Fig. 3B). The conservative strategy adopted by isohydric *Q. acutissima* could ensure the survival and adaptation under drought stress [3].

### Carbohydrate allocations dealing with drought mortality after an abrupt precipitation

During the successive drought stress, the non-structural carbohydrate (NSC) content of *Q. acutissima* seedlings stayed relatively stable. Only an abrupt precipitation occurred after total stomatal closure induced aboveground NSC limitation, and meanwhile, height growth was maintained. Unlikely, both above and belowground NSC limitation were observed in *R*. *pseudoacacia* seedlings, and only the growth of height were limited (Figs. 6, 7). This reflected that *R*. *pseudoacacia* seedlings prioritized growth (long-term carbon storage) through sacrificing NSC storage ("active" carbon storage), and the resource allocation strategy adopted by them could ensure long-term development and survival when undergo resource constraints [28, 35]. However, if NSC storage was below a certain threshold, seedlings may die due to carbon starvation [17]. Carbon starvation might be unfavourable when precipitation occurred again, where seedlings recovery calls for plenty of NSC storage to regrow new tissues or to resprout [32]. Previous studies also showed that trees with very low levels of NSC storage were more likely to die even under favourable environment [22, 33].

The *Q. acutissima* seedlings were prone to keep the NSC pool relatively stable. As the net photosynthetic rate decreased, the proportion of NSC components in most tissues undergoes significant changed, and starch gradually transformed into soluble sugars to maintain normal physiological activity. The strategy of maintaining a relatively stable NSC pool was vital for stress responses and faster recovery after stress, as carbohydrates play a crucial role in supporting and maintaining the metabolic processes of tree seedlings [14]. Specifically, soluble sugars are important regulatory factors in plants, which can regulate osmotic pressure and maintain normal plant life activities under stress. As an inactive storage substance, starch can be converted into soluble sugars under certain conditions to maintain plant survival. When rehydration occurs, the NSC pool could respond rapidly to support the regrowth of new tissues, and provide carbohydrates for basic metabolic activity before new leaves could assimilate enough carbon [34, 29]. The strategy of maintaining NSC pool stable triggered a preferential utilization of carbon reserves in sink tissues, thereby enhanced their ability to adapt and recover from the drought conditions.

## Conclusion

In summary, *R*. *pseudoacacia* seedlings adopted a strategy of sacrificing leaves in response to stress, resulting in the lowered production of photosynthetic assimilates and ultimately leaded to a decrease in non-structural carbohydrates (NSC). In contrast, *Q*. *acutissima* maintains the integrity of leaves by reducing respiratory consumption in response to drought stress, thereby ensured the stability of NSC pool. As an anisohydric species, *R*. *pseudoacacia* exhibited an extravagant strategy with efficient water transport, supporting photosynthetic assimilation and growth capabilities, but its resistance to embolism was relatively weak. As an isohydric species, *Q*. *acutissima* adopted a resource-saving strategy with higher hydraulic safety than* R*. *pseudoacacia.* Given the scenario of progressively rising water stress, if the stomatal regulation mechanism fails to effectively sustain the water equilibrium within plants, the plants may resort to a protective survival approach. Specifically, they will selectively "relinquish" relatively less-constructed organs, including leaves and fine roots. This approach is intended to mitigate water loss and safeguard the hydraulic integrity of the stems, which aligns with the hydraulic vulnerability segmentation hypothesis (Pivovaroft et al., 2014), thereby exhibiting the adaptability and survival acumen of plants in challenging environment. Therefore, if drought stress were severe, *R*. *pseudoacacia* may die of hydraulic failure, while *Q*. *acutissima* may outlived* R*. *pseudoacacia* seedlings. However, under prolonged drought conditions, *Q*. *acutissima* may eventually die due to the depletion of NSC, which may adversely affect the re-sprouting after drought stress relief. We thus propose that it is reasonable to highlight the need to consider multiple hydraulic strategies when evaluate and predict the effect of extreme events. Hence, we recommend that ecologists consider the diversity of ecological strategy in species selection during plantation, which could enhance the response capacity to global climate change.

## Data Availability

Data will be made available on request through the corresponding author.
